# Host-microbiome interactions regarding peri-implantitis and dental implant loss

**DOI:** 10.1186/s12967-022-03636-9

**Published:** 2022-09-23

**Authors:** Carlos Henrique Alves, Karolayne Larissa Russi, Natália Conceição Rocha, Fábio Bastos, Michelle Darrieux, Thais Manzano Parisotto, Raquel Girardello

**Affiliations:** 1 Laboratório de Microbiologia Molecular E Clínica, Programa de Pós-Graduação Em Ciências da Saúde, Universidade São Francisco, 218, São Francisco Ave., Bragança Paulista, São Paulo Zip code: # 12916900 Brazil; 2 Universidade Cruzeiro Do Sul, São Paulo, Brazil

**Keywords:** Oral microbiome, Peri-implantitis, Dental implants, Biofilm, Oral diseases

## Abstract

In the last decades, the ortho-aesthetic-functional rehabilitation had significant advances with the advent of implantology. Despite the success in implantology surgeries, there is a percentage of failures mainly due to *in loco* infections, through bacterial proliferation, presence of fungi and biofilm formation, originating peri-implantitis. In this sense, several studies have been conducted since then, seeking answers to numerous questions that remain unknown. Thus, the present work aims to discuss the interaction between host-oral microbiome and the development of peri-implantitis. Peri-implantitis was associated with a diversity of bacterial species, being *Porphiromonas gingivalis, Treponema denticola* and *Tannerella forsythia* described in higher proportion of peri-implantitis samples. In a parallel role, the injury of peri-implant tissue causes an inflammatory response mediated by activation of innate immune cells such as macrophages, dendritic cells, mast cells, and neutrophils. In summary, the host immune system activation may lead to imbalance of oral microbiota, and, in turn, the oral microbiota dysbiosis is reported leading to cytokines, chemokines, prostaglandins, and proteolytic enzymes production. These biological processes may be responsible for implant loss.

## Background

In the last decades, significative advances have been occurred through research about dental implants. Even though Branemark osteointegration system was not successful, it generated elevated amount of data that lead to improvements in implant techniques [1]. It was at the turn of the millennium that a great revolution took place when the surgical and biomaterials areas brought greater clarity and the desired safety in the procedures performed. The rehabilitation of missing teeth is present in the history for a long time, but not long ago, intraosseous implants started to be installed in rough surfaces, causing no trauma to the adjacent bone and the prosthesis projected for longevity intentions [2]. Due to the progress made, including the aesthetic-functional area of implants, there is an increase in the demand for these procedures in oral rehabilitation. According to Thoma et al. [3], the reasons for this success are studies in implant surface treatments, implant design, material, and techniques, occurring with the increased demand for treatments with dental implants. In addition, the improvement of oral microbiome knowledge has been looking for solutions to problems as biofilm formation and selection of pathogenic bacteria, to avoid infections episodes. This literature review aims to describe the factors involved in the implant survivor by microbiological perspective and immune host response.

### The oral microbiome of peri-implantitis

The Human Oral Microbiome Database (www.homd.org) include, in the adult individual, approximately 772 prokaryotic species, where 70% is cultivable species, and 30% belong to the uncultivable class of microorganisms. Out of 70% culturable species, 57% have already been assigned to their names. Recently, the microbiome sequencing techniques has been used more frequently, allowing non-cultivable species to have been increasingly described.

**Fig. 1:**
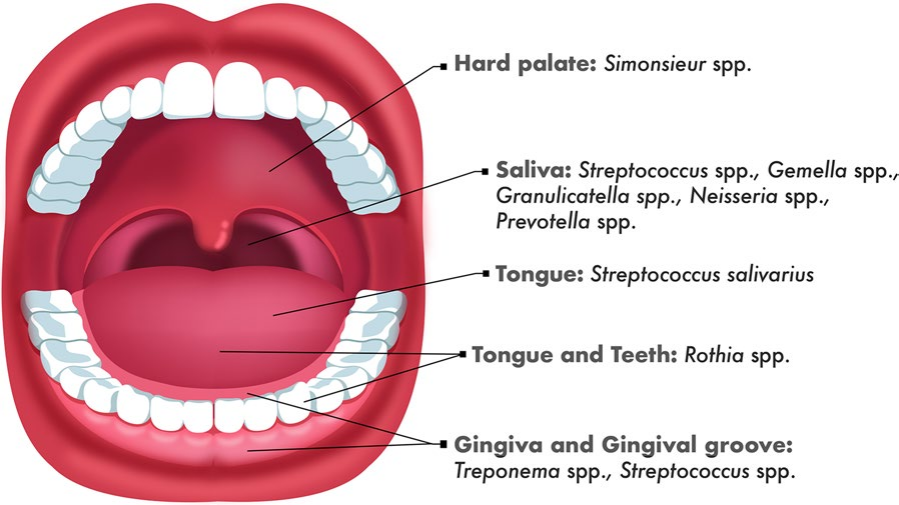
Oral microbiota distribution according to their metabolic and biochemical characteristics. Although there is an overlap of all species in all oral sites, species of *Streptococcus* spp.*, Gemella* spp.*, Granulicatella* spp.*, Neisseria* spp., and *Prevotella* spp. are found more frequently in the saliva. In other hand, it appears that bacteria that are located on the hard palate are not primarily the same as those present on the tongue. *Rothia* spp. typically colonize the tongue or tooth surfaces, *Simonsieur* spp. colonizes only the hard palate, *S. salivarius* mainly colonizes the tongue, and *Treponema* spp. is typically restricted to the gingival and subgingival tissue

Diversity of species colonization occurs into oral cavity, according to the region or even the conditions of this cavity, and the microorganisms are distributed according to their metabolic and biochemical characteristics. The salivary microbiome is essentially composed of a mixture of all microbial sites. Although there is an overlap of all species in all oral sites, species of *Streptococcus* spp.*, Gemella* spp.*, Granulicatella* spp.*, Neisseria* spp., and *Prevotella* spp. are found more frequently in the saliva [4]. On the other hand, it appears that bacteria that are located on the hard palate are not primarily the same as those present on the tongue. *Rothia* spp. and *S. salivarius* mainly colonizes the tongue or tooth surfaces, *Simonsieur* spp. colonizes only the hard palate, and *Treponema* spp. is typically restricted to the gingival and subgingival tissue [5]. Oral healthy gingival tissue presents more commonly *Streptococcus* species, but, in the gingival disease’s tissue, *Streptococcus* spp. are replaced by *Treponema* spp. The *Treponema* spp. are described as one of the first pathogens in the gingival tissue (Fig. 1) [6].

Furthermore, bacteria are guided through adhesin binding receptors. Some receptors come from salivary proteins and through the affinity by bacteria initiating the biofilm accumulation. These receptors are derived from salivary components such as proteins rich in proline and serine, which are submitted to changes in conformation when adhered to surfaces such as the tooth where there is a specific interaction with a strong affinity. Issues such as adherence and functionality are some of the factors that help the bacterial complex to form the most convenient biofilm [7, 8].

It is quite usual the concern about the bacteria involved in the infection and biofilm formation, probably due to 16S sequencing methodology used by the most of studies. However, metagenomic studies have revealed that fungus has a large participation in biofilm establishment. *Candida albicans*, *C. guilliermondii*, *C. glabrata*, *Rhodotorula* spp. and *Trichosporon* spp. are identified in oral microbiota and the most yeasts identified had ability to form biofilms and presented resistance to the antifungal agents [9]. In addition, diverse *Trichosporum* species are also able to form biofilms, such as *T. asahii*, *T. asteroides*, *T. cutaneum* and *T. mucoides* [10].

The investigation of the oral microbiota in pathogenic situations is necessary together with the advent of implantology to identify causes and seek for relevant solutions, so that patients do not lose the functionality, whether esthetic or bio-mechanical, of the installed implants caused by accumulation of biofilm and consequent loss of peri-implant bone support.

**Fig. 2:**
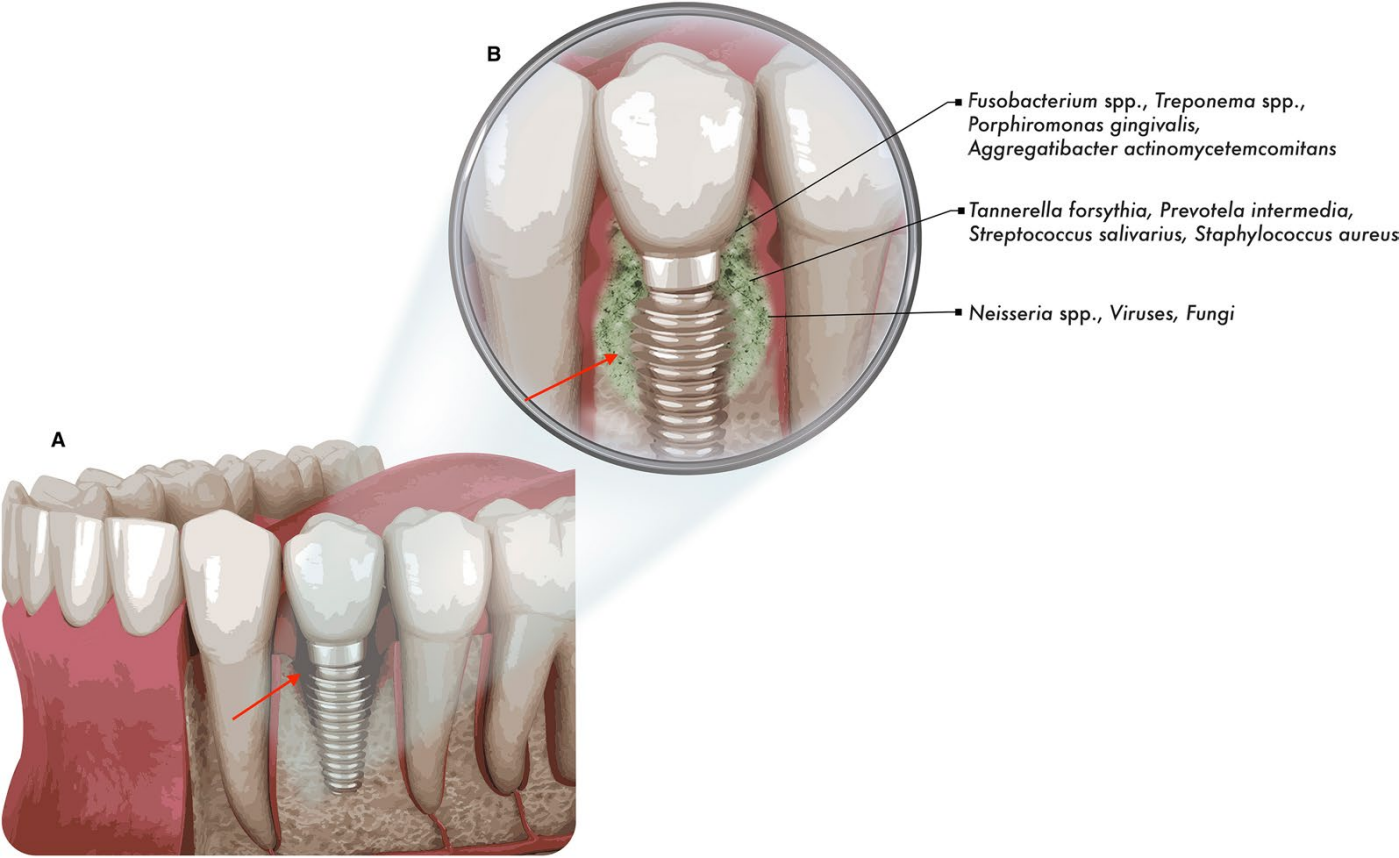
Peri-implantitis causing bone loss, exposition of dental implant, and biofilm formation. **A** Schematic illustration of bone loss around implant (red arrow). **B** Red arrow correspond to implant exposition for bacterial colonization, after bone loss. The area around implant resulting from bone loss is filled by complex biofilm community (green) formed by diversity bacterial, fungal, and virous species, distributed according to nutritional and biochemical needs. Bacterial species described in higher proportion of peri-implantitis are responsible for initiation of community formation and, in an opportunistic way in the infectious process, other species of bacteria, fungi and viruses are also part of this biofilm complex

One of the main causes of implant loss is due to peri-implantitis incidence. Peri-implantitis is caused by the biofilm accumulation that occurs in the tissues around dental implant, causing inflammation in the peri-implant mucosa, followed by progressive loss of the supporting bone (Fig. 2) [11]. Peri-implantitis is associated with a diversity of bacterial species, such as *Tannerela forsythia*, *Porphiromonas gingivalis, Aggregatibacter actinomycetemcomitans*, *Prevotella intermedia* and *S. salivarius* [12, 13]. *P. gingivalis, Treponema denticola* and *Tannerella forsythia* are described in higher proportion of peri-implantitis samples [13]. Some studies also indicated the presence of pathogens such as *Staphylococcus aureus* and *Pseudomonas aeruginosa,* acting in an opportunistic way in the infectious process; fungi and viruses are also part of this biofilm complex [14, 15].

In a temporal analysis is possible observe that Firmicutes phylum increase during the maturation of peri-implant plaque, and a decrease of *Neisseria* spp. and *Porphyromonas* spp*.* detection is observed after periimplantitis establishment [16]. Initially the peri-mucositis appears and then the peri-implantitis can develop later. Peri-implant mucositis resembles gingivitis in natural teeth; it appears that there is an increase in both richness and diversity of the subgingival microbiome during gingivitis, and while richness remains high in periodontitis because no species are lost during the shift, it seems that some species become dominant (i.e., increase in proportion) in periodontitis associated communities, decreasing community evenness, and therefore reducing the overall diversity compared with gingivitis. Gingivitis and peri-mucositis microbiome are similar in etiology [17]. Still, peri-implant mucositis is defined as an inflammation of the soft tissues (i.e., peri-implant mucosa) around the implant and presents clinically with bleeding upon simple probing [18]. Through peri-implant mucositis, peri-implantitis can develop or not. There are etiological similarities between the biofilm found in periodontitis and peri-implantitis; however, in the second, differentiated bacteria were also identified. According to Klinge et al. [19], in terms of clinical appearance and pathogenesis, it is accepted that both have a common etiology: oral dysbiotic biofilm. However, the predominant bacteria in peri-implantitis comparing with healthy implants are *Fusobacterium* spp. *and Treponema* spp.; but, in peri-mucositis it was mostly colonized by *Rothia* spp. and *Streptococcus* spp [20]. In summary, peri-mucositis is more associated with common periodontal pathogens, while peri-implantitis harbor differentially abundant communities [21]. A study from Ghensi et al. suggest that *Fusobacterium nucleatum* appears to be the first specie that has increased abundance along the mucositis–peri-implantitis axis and result in more strongly peri-implantitis at later stages of the disease. In this way, the knowledge of the oral microbiome based on a phylogenetic database to be important to identify the pre- and post-surgical bacterial niche and develop studies for possible prevention or even prophylactic measures to reduce the loss of dental implants caused by peri-implantitis.

#### The dental implant composition and biofilm formation

Associated to microbiota influence, the material compounds the dental implant also develop important role in the osteointegration process and may act as originator to biofilm formation. On the other hand, their composition is also responsible for greater or lesser bacterial adhesion and consequent biofilm accumulation (Table 1). Bacterial infections are common cause of dental implant failure and due to challenging treatment, occasionally is necessary the tooth removal [23]. Similar to the biofilm formation on natural teeth, bacterial colonization occurs within minutes after the implantation procedure and throughout the life cycle of an implant, therefore it is necessary an implant surface treatment. Surface roughness, which directly affects both osseointegration and biofilm formation, is the primary target for all kinds of surface modifications. However, it is difficult to determine because a surface roughness of at least 1–1.5 µm is needed to achieve a good bone fixation, while the bacterial retention threshold is 0.2 µm, above which an increase in bacterial accumulation occurs. Designing an optimal implant surface, there must be a fine balance between antimicrobial activity and the desired osteoconductive properties. The balance is difficult to achieve as greater surface roughness promotes firmer bone fixation but is directly proportional to bacterial retention, which may promote biofilm formation in the long run [24].

**Table 1:** The dental implant composition and their ability to prevent biofilm formation

Implant composition	Advantages	Disadvantages	References
Titanium plus Apatite Hydroxide	•Good biocompatibility; •High resistance; •Good biosecurity	•Insufficient soft tissue integration; •Vulnerability to biofilm accumulation; •Contribute to oral microbiome dysbiosis; •Induces peri-implantitis development	[26–29]
Zirconium Dioxide	•Excellent biocompatibility; •Good tissue integration inducing low bone reabsorption; •Low affinity to bacterial biofilm	•Weak material; •Frequently fracture	[30–33]
Titanium implants coated with zirconia	•Reduced adhesion of *S. mutans* and *P. gingivalis*	More studies are necessary	[34]
Zirconia plus TiO_2_ coverage	•Favorable for osteogenic effects	More studies are necessary	[34, 35]
Ceramic-based alternatives	•Anti-inflammatory and antimicrobial properties; •When associated with bio-glass, demonstrate reduction of methicillin-resistant *S. aureus* (MRSA) growth	•The processing and shaping them is demanding, and thus accessible design options are limited	[25]
Nanostructures-based alternatives	•Better osteointegration; •Good surface porosity, roughness, and wettability; •Included bioactive components; •Increased osteoblast proliferation; •Low bacterial adhesion; •Low biofilm maturation of pathogenic species *P. gingivalis*, *T. denticola* and *T. forsythia* •Decrease in pathogenic species *S. aureus* and *P. aeruginosa* viability	More studies are necessary	[23, 36–39]
Polyetheretherketone (PEEK)	•Mechanical and physical properties like bone and dentin; •Wettability and nano-roughness demonstrating bactericidal and/or anti-adhesive effect on biofilm biomass from *Streptococcus oralis*	More studies are necessary	[40, 41]
Carbon fiber-reinforced PEEK (CFR-PEEK)	•Reduced lateral stress on implants as well as crestal bone loss	•No microbiological studies were performed for this structure until now, to verify the biofilm formation	[42]

Titanium has been used for dental implants since the 1960s and is one of the most biocompatible materials, biologically inert and highly resistant to corrosion due to spontaneous formation of a titanium oxide (TiO2) film on its surface, which separates the metal from its environment, providing bio security against local infections [25]. A layer of apatite hydroxide is applied to the implant surface to obtain better osteoconductive, helping in the osteointegration process [26]. So, titanium is still the most widely used dental implant material. However, insufficient soft tissue integration and vulnerability to biofilm accumulation are described for this implant composition [27]. An in vitro study from Souza et al. [28], revealed that titanium products, especially ions, have potential to change the microbiological composition of biofilms formed on its surfaces. The authors affirm that the presence of titanium products around dental implants may contribute to oral microbiota dysbiosis and, in consequence, to peri-implantitis development.

The zirconium dioxide (zirconia) has earned its place to substitute titanium in implant manufacturing due to its excellent biocompatibility, tissue integration inducing low degree of bone resorption, and low affinity to bacterial biofilm, besides its biomechanical properties [29–31]. However, according to Sanon et al. [32], the main concern about this material due to technical failures occurred like fracture of the material, that is the reason of titanium remains the best material; so, the association of both titanium and zirconium dioxide material is one option to avoid this effect. According to Tang et al. [33], modifying the surface of zirconia with a TiO_2_ coating might be favorable for osteogenic effects. In addition, Jo et al. [34] showed that, when the surface of titanium implants coated with zirconia via atomic layer deposition, the level of *S. mutans* and *P. gingivalis* adhesion is reduced regardless of the presence of zirconia crystal phases deposited on the surface.

Ceramic-based alternatives are promising to avoid metal surfaces use; however, according to Matter et al. [23] the processing and shaping them is demanding, and thus accessible design options are limited. These authors develop nanostructured implant coatings based on such multi-metal oxide nanohybrid building blocks tailored to both hard and soft tissue healing. Ceria has proven anti-inflammatory and antimicrobial properties and their association with bioglass demonstrate a reduction of methicillin-resistant *S. aureus* (MRSA) growth [23].

Currently, nanostructures have been suggested as solution for better osteointegration e success of implants. Nanostructured implants have an increased surface area, allowing for better adsorption of proteins and improved attachment of cells to the implant. To improve the implant surface porosity, roughness, and wettability, and to incorporate bioactive components, a wide spectrum of nanocoating techniques has been utilized on dental implants [23]. Associated to increased osteoblast proliferation, the titanium nanoparticles surface appears demonstrate low bacterial adhesion and low biofim maturation of pathogenic species *Porphyromonas gingivalis*, *Treponema denticola* and *Tannerella forsythia,* after 30 days of exposition, compared with other surfaces composition [35]. Another study from Bright et al. [36] showed a decrease in pathogenic species *Sthapylococcus aureus* and *Pseudomonas aeruginosa* viability in the 2 μm layer furthest from the nanostructured surface from titanium nanostructured Ti6Al4V.

Regarding chemical modifications, nano-level modifications are achieved to increase the surface’s hydrophilicity and thus promote osseointegration while reducing hydrophobic bacterial adhesion. Moreover, more robust, and direct stimulation of osseointegration and mitigation of biofilm formation can be accomplished by specific biological modifications. For example, growth factor-coating is known to enhance osseointegration, while antibacterial agent-coating directly combats bacteria and enhances implant properties [24].

According to the Heo et al. [37], gold nanoparticles have been successfully tested immobilizing titanium implant surfaces as osteo inductive agent for fast osseointegration. The effectiveness in vitro and in vivo were investigated and, in both experiences, they ensure that gold nanoparticles can be useful as osteo-integration, inducing dental implants to form osteo interface also helping the maintenance of a new bone formation as well. Baumer et al. [38] affirms on her studies that miR-335-5p/Lipidoid nanoparticles coated on titanium surfaces has shown a transfection efficiency, cell adhesion, proliferation, and osteogenic activity of the bone-implant interface, therefore it might be used as a new methodology to improve the osteogenic capacity of biomedical vcom D'Ercole et al. [41] demonstrate that wettability and nano-roughness of PEEK can significantly affect the concentration of bacteria Colonies Formation Units (CFUs) and biofilm biomass from *Streptococcus oralis*, demonstrating bactericidal and/or anti-adhesive effect. In addition, a carbon fiber-reinforced PEEK (CFR-PEEK) has been evaluated to replace titanium in manufacture of implants. According to Tamarakar et al. [42] the amount of stress generated within the bone in the case of the CFR PEEK implants with different restorative crowns was smaller in comparison with the titanium implants in oblique loading. This could help reduce lateral stress on implants as well as crestal bone loss. No microbiological studies were performed for this structure until now, to verify the biofilm formation.

Recent studies bring new probable future solutions to rehabilitate missing teeth. According to Galler et al. [43], stem cells have been studied for teeth regeneration. Many successful engineering initiatives related not only to the whole tooth regeneration but also for enamel, the dentin-pulp complex and periodontal ligament have been developed. This task might enable regenerative development for the dentistry in the future.

Regardless of the composition of the implant, manufacturing failures are generally responsible for less osseointegration and increased bacterial colonization, leading to an increase in peri-implantitis rates. According to Belibasakis et al. [21], biocorrosion of the implant, abutment loosening, prosthetic screen loosening, milled abutment, or prosthetic screw, decemented crown may affect microbial colonization and disease progression.

In addition, there is a straight co-relation between the peri-implantitis development and implant structural manufacturing characteristics as well as prosthetic manufacturing errors which causes surfaces exposure and collaborates for biofilm formation. Peri-implantitis and peri-mucositis are generally identified in patients with non-closed crown edges, loose crown-retained screws, loose abutment screws and broken abutment screws, considered risky reasons associated with the implantation time, and implant position [44]. In addition, prosthesis not well positioned causing difficulties for the appropriate hygiene leading to biofilm formation and a future peri-implantitis [15].

Additional influencing factors for implant loss is the implant type, wider implant diameter, and number of implants per patient [38]. In a study from Zahng et al. [44], peri-implantitis and peri-mucositis occurred at lower rates in the Straumann system, while the Osstem system had higher occurrences, and significant higher incidence was observed in the anterior maxilla area. In addition, Jansson et al. [45] described difference in peri-implantitis and peri-mucositis incidence in the implant surface. The implant regions with highest incidence of moderate/severe peri-implantitis, and consequent bone loss, were mandibular incisor/canine and maxillary incisor/canine; while the occurrence of peri-mucositis was in the regions of maxillary molar, maxillary incisor/canine, and mandibular premolar.

### Host immune system and microbiota interactions in peri-implantitis

Peri-implantitis is a multifactorial condition affecting soft tissue and bone around the implant and is resulting from an imbalanced interaction between the pathogen and the host immune response. This process is characterized by inflammation in the peri-implant mucosa and subsequent progressive loss of supporting bone [46–48]. The host immune system activation may lead to imbalance of oral microbiota. In turn, the oral microbiota dysbiosis is reported leading to cytokines, chemokines, prostaglandins, and proteolytic enzymes production [48]. Microorganisms from oral microbiota are spread in the oral cavity forming communities interacting synergistically. One microorganism act providing a substratum for the attachment and colonization of another. In addition, physical interactions between the microorganisms can modulate gene expression, including survivor advantage or virulence genes. The availability of nutrients and oxygen are some of factors determining for this distribution [49].

The injury of peri-implant tissue causes an inflammatory response mediated by activation of innate immune cells such as macrophages, dendritic cells, mast cells, and neutrophils. The neutrophils promote the release of pro-inflammatory citokines IL-1 and TNF-α, which in turn activate the osteolytic and inflammatory tissue damage observed in peri-implantitis [50]. According to Hashim et al. [51], defects in the number or efficacy of neutrophils predispose individuals to development of periodontal disease. Paradoxically, neutrophil activity, as part of a deregulated inflammatory response, appears to be an important element in the destructive disease process. The absence of CXCR-2 neutrophil receptor in gingival tissue significant was associated with changes in the local microbiome, resulting in an increase of periodontal disease. This effect demonstrates the important role of neutrophils in balancing the oral microbiota. Furthermore, the presence of active CXCR-2 neutrophil receptors was able to reestablish the gingival tissue microbiota [51].

**Fig. 3:**
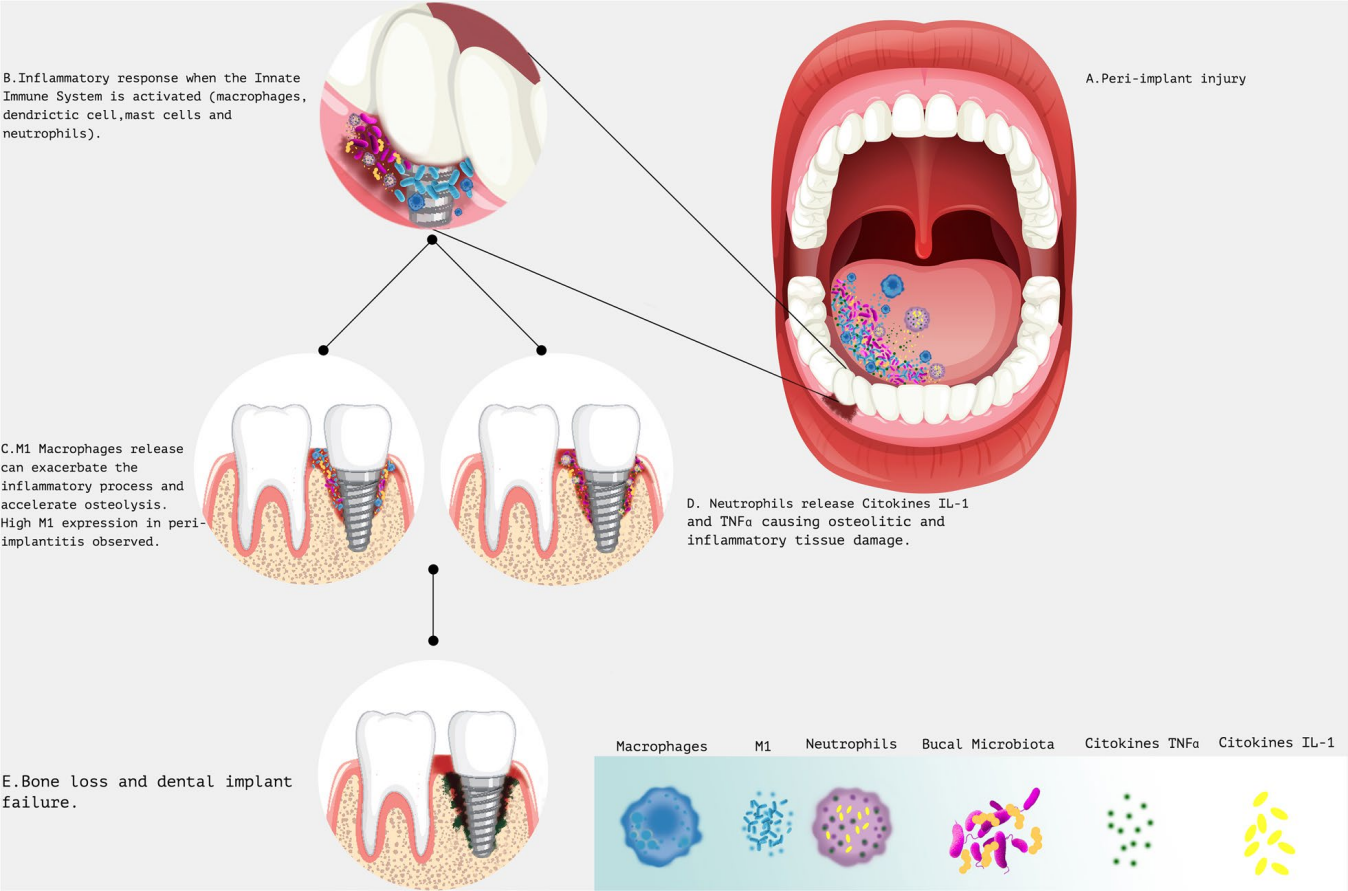
Peri-implantitis is a multifactorial condition affecting soft tissue and bone around the implant and is resulting from an imbalanced interaction between the pathogen and the host immune response. The inflammation in the peri-implant mucosa and subsequent progressive loss of supporting bone injury of peri-implant tissue causes an inflammatory response firstly mediated by activation of innate immune cells such as macrophages, dendritic cells, mast cells, and neutrophils, that induces inflammatory process, leading both microbiota disrupting and osteolysis process inducing. The neutrophils promote the release of pro-inflammatory citokines IL-1 and TNF-α, which in turn activate the osteolytic and inflammatory tissue damage observed in peri-implantitis, while macrophages release participate in the inflammation exacerbation and consequent accelerating osteolysis. The bone loss creates an environment to biofilm formation

Meanwhile, macrophages might have a binary role in directing the implant failure or success, depending on their phenotype [50]. M2 macrophages could lead to osseointegration and effective wound healing, while the M1 macrophages could exacerbate the inflammatory process and accelerate osteolysis leading to dental implant failure. Macrophage polarization has been observed in peri-implantitis lesions. Increased populations of M1 macrophages on peri-implantitis samples were observed when compared to periodontal disease samples. Advanced peri-implantitis cases expressed a significantly higher M1 profile when compared with M2 expression (Fig. 3) [52, 53]. Contrasting these observations, a machine learning-assisted immune profile was designed by Wang et al. [54], where patients at low risk of developing peri-implantitis exhibited elevated M1/M2-like macrophage ratios, higher frequencies of regulatory T cells and lower B-cell infiltration. The association of increased M1 population and low risk of peri-implantitis could be explained by the promotion of Th1 responses which are more effective at controlling pathogens that may contribute to disease progression. However, more studies by using this technology are necessary to confirm this founds.

Furthermore, the low-risk immune profile is also characterized by enhanced complement signaling and higher levels of Th1 and Th17 cytokines. An increased expression of complement components correlated with improved outcomes. In addition, *F. nucleatum* and *Prevotella intermedia* were significantly enriched in high-risk individuals, compared with the low-risk group. *F. nucleatum* is also reported inducing human beta defensins that act as antimicrobial and chemotactic agents, due to a lipo-protein FAD-I (*Fusobacterium* Associated Defensin Inducer) associated with their outer membrane [55]. The combination of complement activation and increased cytokine production could contribute to limit the microbial burden on the oral cavity of the patients, resulting in reduced risk of peri-implantitis in this group.

Saremi et al. [47] evaluated the influence of immune gene polymorphisms on the development of peri-implantitis and revealed that allele and genotype frequencies of IL-10 ─ 819 C/T, IL-10 ─ 592 C/A, and IL-1ß + 3954 C/T, significantly differed between diseased and healthy patients, indicating that these specific gene polymorphisms may play a role in the pathogenesis of peri-implantitis. In addition, several studies evaluating gene expression showed several pathways regulating in the inflammatory response in the peri-implantitis. Treg and TH17 cells influence the inflammatory process in periodontal diseases and appears to play an essential role in the destruction of the peri-implant tissues [48]. *P. gengivalis* is one of major pathogen-causing peri-implantitis and studies demonstrate that this pathogen may active several pathways for immune system response, including LOX-1 and TLR4 receptors, that induces the production of osteopontin, involved in differentiation of odontoblast-like cells, and Wnt5a, a ligand of Wnt signaling pathways involved in leukocyte infiltration and cytokine/chemokine production. In addition, IL-1β, MMP2 and MMP9 production in response to *P. gingivalis* in THP-1 macrophages was also dependent on LOX-1 [46, 56, 57].

Another factor that has been linked to inflammation and tissue damage is the excess of cement on the prosthetic crown, that is associated with bacterial accumulation [58]. It also promotes tissue inflammation since it is recognized as a foreign body by the host immune system. The immune activation and increased bacterial rates are associated with histological changes in the tooth and lead to lower survival of local osteoblasts.

Li et al. [59] demonstrates that a fine tuning of osteoclast-osteoblast balance is required for a perfect synchronization of bone resorption and formation, to maintain efficient bone remodeling and bone homeostasis. By contrast, activation of the inflammasome during bacterial infection may leads to bacterial spreading or even an uncontrolled bone destruction, which is very common in periodontitis, periapical periodontitis, peri-implantitis and other related conditions. This uncontrolled inflammasome activity may cause alveolar osteolysis by activated macrophages, monocytes, neutrophils, and adaptive immune cells like T helper 17 cells. This whole immune response causes an increase in osteoclasts and concomitant decrease of osteoblasts. In addition, osteocytes play an important role in alveolar bone loss, since they respond to inflammatory changes by secreting molecules that affect bone resorption and formation, causing bone loss [60].

### Potential preventions or therapies for microbiological-driven peri-implantitis

The current antibiotic resistance rates associated with microbiota dysbiosis caused by antibiotic and antiseptic usage, probiotics have been suggested as option for peri-implantitis treatment. Probiotics are defined as living and viable microorganisms which, when administered in adequate quantities, confer benefits to the organism [61]. Probiotics are considered a safe and useful tool, since reduces the immunogenicity of microbiotas by improve the balance of the host microorganisms, inhibiting pathogens. In addition, the host balance result in immune homeostasis, and consequent decreasing of proinflammatory cytokines (Table 2) [13].

**Table 2:** Probiotic-based therapy for prevention of peri-implantitis and peri-mucositis process

Probiotic composition	Efficacy	References
*Lactobacillus* species	•Inhibit the pathogenic bacteria *S. mutans*, *A. actinomycetemcomitans*, *P. gingivalis* and *P. intermedia;* •Strongest antimicrobial activity was associated with *L. paracasei*, *L. plantarum*, *L. rhamnosus* and *L. salivarius*	[60]
*L. reuteri*	•Slight decreasing of peri-implantitis rate; •Reduction of periodontal and peri-implantitis related species, as *P. gingivalis;* •Prevention of inflammation, reducing mucositis process; •Clinically effective in terms of pocket depth reduction in this peri-implantitis treatment, but without reaching baseline levels	[59–64]
*S. salivarius*	•Bacteriocin produced by *S. salivarius* inhibit the *quorum-sensing* signals and reducing the *S. intermedius* biofilm formation in titanium implant surface; •May be ineffective in peri‐implant disease treatment, when caused by *C. albicans* pathogen	[65, 66]

*Lactobacillus* species have been used for years to balance gut and vaginal microbiotas, and currently is suggested also to oral microbiota. In a study from Kõll-Klais et al. [62] the most prevalent *Lactobacillus* species in oral microbiota from healthy individual were *L. gasseri* and *L. fermentum,* while in chronic periodontitis patients, *L. plantarum* was more frequent. In in vitro tests, *Lactobacillus* spp. was able to inhibit the pathogenic bacteria *S. mutans*, *A. actinomycetemcomitans*, *P. gingivalis* and *P. intermedia*. Strongest antimicrobial activity was associated with *L. paracasei*, *L. plantarum*, *L. rhamnosus* and *L. salivarius*.

*L. reuteri*-based probiotics are used for gut microbiota balance and was suggested for oral microbiota to peri-implantitis treatment. Two studies showed low decreasing of peri-implantitis rate; however, there was a reduction in the number of periodontal and peri-implant causing species, as *P. gingivalis* [61, 63]. On the other hand, after treatment with the probiotic *L. reuteri* in patients with implants presenting mucositis, the clinical parameters improved, and the cytokine levels decreased, suggesting a preventing role of *L. reuteri*-based probiotics [64]. Though not always achieving significance, studies show difference in the depth of the probing in the treatment of peri-implantitis, when using *L. reuteri* probiotic, which can be clinically effective in terms of pocket depth reduction in this treatment [65, 66]. Until now, studies demonstrated that the *L. reuteri*-based probiotics appears to be not so effective solution for peri-implantitis diseases; however, more studies evaluating other probiotics composition need to be performed to make available an associated therapy and reduction of antiseptic and antibiotic usage.

A novel proposal to *S. salivarius*-based probiotic in reduction of biofilm formation in implant was demonstrated by Vacca et al. [67]. The authors demonstrated an interaction between bacteriocin produced by *S. salivarius* inhibiting the quorum-sensing signals and reducing the *S. intermedius* biofilm production in titanium implant surface; so, this probiotic could be considered in non-surgical therapy to prevent biofilm-related implant diseases. On the other hand, a study from Martorano-Fernandes et al. [68] suggests that the use of *S. salivarius* as a probiotic would be ineffective in peri‐implant disease treatment, when caused by *C. albicans* pathogen.

There is a lack of diversity about probiotic composition for peri-implantitis therapy, so, more studies are necessary to invested in an adjuvant and non-medicated solution, to avoid as much as possible as the imbalance of the oral microbiota.

## Conclusion

Obtaining knowledge about the specificity of the oral microbiome shows the complexity and difficult task against oral infections. The host-microbiome interactions may contribute for the periimplantitis development. According to this review, we have observed the oral microbiome dysbiosis may be the starting point for an unbalanced concentration of different bacteria species, therefore it collaborates for a strong biofilm formation due to its bacteria diversity. This process associated with some implant structural manufacturing and prosthetic mistakes may facilitate and become easier the biofilm formation. The host immune system has an important role in the oral dysbiosis, leading to citokines, chemokines, prostaglandins, and proteolytic enzymes production in the oral microbiota, which may induce unusual bacteria interactions causing bone loss derivate from peri-implantitis development, and possible implant loss. Probiotics are considered a safe and useful tool, since reduces the immunogenicity of microbiotas by improving the balance of the host microorganisms, inhibiting pathogens. The identification of the microbiome, as well as its quantification, are fundamental data necessary for future behavioral analysis of these colonization and their conveniences in the creation of biofilms and their interactions relating to peri-implantitis development.

## Data Availability

Not applicable.
